# Efficacy of Propolis on the Denture Stomatitis Treatment in Older Adults: A Multicentric Randomized Trial

**DOI:** 10.1155/2017/8971746

**Published:** 2017-03-15

**Authors:** Gisela de M. S. Pina, Erica N. Lia, Andresa A. Berretta, Andresa P. Nascimento, Elina C. Torres, Andrei F. M. Buszinski, Tatiana A. de Campos, Eduardo B. Coelho, Vicente de P. Martins

**Affiliations:** ^1^UniEvangélica University Center, Anápolis, GO, Brazil; ^2^University of Brasilia (UnB), Brasilia, DF, Brazil; ^3^Laboratory of Research, Development and Innovation, Apis Flora Indl. Coml. Ltda., Ribeirão Preto, SP, Brazil; ^4^University of São Paulo (USP), Ribeirão Preto Medical School, Ribeirão Preto, SP, Brazil

## Abstract

Our hypothesis tested the efficacy and safety of a mucoadhesive oral gel formulation of Brazilian propolis extract compared to miconazole oral gel for the treatment of denture stomatitis due to* Candida* spp. infection in older adults. Forty patients were randomly allocated in a noninferiority clinical trial into two groups. The control group (MIC) received 20 mg/g miconazole oral gel and the study group (PROP) received mucoadhesive formulation containing standardized extract of 2% (20 mg/g) propolis (EPP-AF®) during 14 days. Patients were examined on days 1, 7, and 14. The Newton's score was used to classify the severity of denture stomatitis. The colony forming unity count (CFU/mL) was quantified and identified (CHROMagar* Candida*®) before and after the treatment. Baseline characteristics did not differ between groups. Both treatments reduced Newton's score (*P* < 0.0001), indicating a clinical improvement of the symptoms of candidiasis with a clinical cure rate of 70%. The microbiological cure with significant reduction in fungal burden on T14 was 70% in the miconazole group and 25% in the EPP-AF group. The EPP-AF appears to be noninferior to miconazole considering the clinical cure rate and could be recommended as an alternative treatment in older patients.

## 1. Introduction

Candidiasis is the most common fungal infection that occurs in the mouth [1, 2]. The microorganisms involved belong to the genus* Candida *sp. and the species most commonly found at sites of infection is* Candida albicans*. However, less frequently other species are found, such as* C. tropicalis*,* C. glabrata*,* C. krusei, C*.* parapsilosis, C. guilliermondii, and C. dubliniensis* [2, 3].* Candida *sp. is a commensal organism, and its transformation into opportunistic pathogen occurs due to fungal pathogenicity mechanisms such as epithelial adherence, morphogenesis, production of hydrolytic enzymes, and phenotypic changes. The immune defense of the host and local defense responses are actively involved in the resistance to the fungus pathogenic mechanisms and the development and progress of infection [3, 4]. Thus, immunosuppression, (HIV infection, corticosteroid therapy, and chemotherapy), diabetes, premature birth, prolonged use of antibiotics, associated with local factors such as hyposalivation, use of inhaled corticosteroids, poor oral hygiene, and continuous use of removable dentures (RD) predispose to the occurrence of oral candidiasis [1].

Among the various clinical forms, there is chronic atrophic candidiasis, known as denture stomatitis, which is characterized by erythematous lesions located mainly in the upper palate, which contact the denture base. The prevalence of the disease among top RD users presented by the literature is 58–88% [1, 2, 5]. The presence of cracks or porosities in the denture acrylic resin acts as fungi reservoirs [1, 2, 6, 7].

Usually, denture stomatitis is treated with antifungals (miconazole, nystatin, and fluconazole), but studies show cases of treatment failure and rapid recurrence after discontinuation of treatment, especially if not accompanied by proper prosthesis hygiene [8]. Drugs resistance mechanisms and antifungal toxicity are also observed [6, 9–11]. Thus, natural products, such as propolis, are gaining prominence. This product is a resinous substance derived from exudates of Brazilian biodiversity, mixed with floral sap, salivary secretions of bees, wax, and pollen.

In previous studies, the safety of standardized extract of propolis (EPP-AF) has been demonstrated, showing that it is a noncytotoxic product and has no mutagenic potential with either oral or topic use [12, 13]. Moreover, EPP-AF has anti-inflammatory [13, 14], antiulcer [15], wound healing, antimicrobial [16, 17], and fungicidal activities [11, 18–21]. Its properties justify the hypothesis to be used as topical treatment of denture stomatitis. The therapeutic effect of alcoholic extract of propolis 20% in denture stomatitis was evaluated in another study, in which there was regression of the lesion in all treated patients; Nystatin was used as positive control [20]. However, the ethanol used as a solvent may irritate the oral mucosa and does not have the mucoadhesive characteristic. In vitro models showed that the 1% alcoholic propolis extract was sufficient to completely inhibit the growth of one million* Candida* cells per millilitre of sample [22]. The efficacy of propolis gel was demonstrated and compared to the use of miconazole oral gel in clinical studies [19, 23].

Thus, the objectives of the study were to test the EPP-AF mucoadhesive formulation efficacy, as well as its safety, acceptability, and anti-*Candida* activity compared to miconazole oral gel (20 mg/g), through the quantification and identification of isolated* Candida* species of the mucosa of the population studied before and after treatments.

## 2. Materials and Methods

### 2.1. Trial Design

This is a multicenter, randomized open-label, parallel arm trial.

### 2.2. Participants

The inclusion criteria were older adults of both sexes, aged over 60 years, using superior complete removable acrylic denture and presenting denture stomatitis. Exclusion criteria were cognitive decline or dementia, and recent use of antibiotics and/or antifungals (in the past 2 months). None of the volunteers had been submitted to radiotherapy after head and neck cancer.

Volunteers were enrolled at the University of Anápolis (UniEvangelica) and the Family Health Center of the Ribeirão Preto Medical School, University of São Paulo (FMRP-USP) between January and June 2016. The volunteers agreeing to participate in the study signed the informed consent form.

Demographic data and medical history were collected through anamnesis on the first day (T0). The volunteers were asked about the duration of use of prosthesis, habits, frequency, and products used for oral hygiene and prosthesis and also about the habit of sleeping with the prosthesis and medical history.

The diagnosis of denture stomatitis was performed through clinical evaluation. After removal of the prosthesis, the subjects underwent an intraoral examination under artificial lighting, on the first day (T0), 1 week after the first examination (T7) and 14 days after the first examination (T14). The hard palate was examined, and the presence of erythematous spots or areas, accompanied or not by papillary hyperplasia, was observed under the pliable area of the prosthesis. In order to classify the lesions, Newton's classification [24] was divided into type I (localized inflammation or points of hemorrhagic petechiae); Type II (more diffuse erythema involving part or all of the area covered by the prosthesis); and Type III (erythema associated with papillary hyperplasia in the area covered by the prosthesis).

### 2.3. Interventions

The volunteers were randomly assigned to 2 groups, the control group (MIC) treated with oral gel containing 20 mg/g miconazole and the propolis group (PROP) treated with a mucoadhesive formulation containing gel with EPP-AF propolis standard extract 2%. The ingredients of the formulation were a standardized extract of propolis (EPP-AF) sorbitol, pectin (polymer) potassium sorbate (preservative), ascorbic acid (acidulant), sweeteners (xylitol and sucralose), aromas (menthol and vanilla), and purified water. EPP-AF was produced and supplied by Apis Flora Industrial and Comercial Ltda (Ribeirão Preto, SP). The extract was standardized using a crude propolis composition obtained from several regions of Brazil, with a predominance of green propolis originated from* Baccharis dracunculifolia* (patent number 0405483-0).

The volunteers were instructed to perform oral and prosthesis hygiene, sprinkle 0.12% chlorhexidine digluconate solution on the prosthesis basis, remove the excess, and apply a thin layer of miconazole oral gel 20 mg/g, or gel with EPP-AF on the inner surface of the prosthesis, 3 times a day for 14 days. All patients were instructed about oral hygiene, prosthetic maintenance, and medication application, and they were instructed to remove the prosthesis during the sleep period and to accommodate it in a container with water. Patients received a preweighed miconazole or EEP-AF gel pipe, a 0.12% chlorhexidine solution vial, and written instructions for use.

To evaluate the fungal burden, mouthwash samples were collected using 20 mL of 0.9% sodium chloride solution for 20 seconds, at days 1 and 14. The mouthwash was dispensed in sterile tube and refrigerated until laboratory processing. The collections followed protocol from previous studies [24–27].

The samples were centrifuged for 15 minutes at 25°C at 3,000 ×g, the supernatant was discarded, and the pellets were resuspended in 1 mL of 0.9% sodium chloride solution. The first dilution of each sample was made in 0.9 mL of 0.9% sodium chloride solution and 0.1 mL of the initial sample, which was 10^−1^ (1 : 10). The second dilution was made by collecting 0.1 mL of the previous dilution (10^−1^) in 0.9 mL of 0.9% sodium chloride solution, 10^−2^ (1 : 100). One hundred microliters of the samples was seeded by spreading on sterile plates containing the Sabouraud dextrose agar (SDA) culture medium (Difco, Detroit, MI, USA) which was prepared according to the manufacturer's instructions and added with 5 mL chloramphenicol 0.1 g/mL to 1,000 mL of the medium. A fourth plaque was seeded by exhaustion in CHROMagar* Candida* medium (CHROMagar Company, Paris, France) for identification of the species and stored in a 37°C stove for 48 h. Then the CFU/mL count and identification of the* Candida* species were made for comparison at the beginning and end of the treatment and comparison between the groups.

The miconazole and EPP-FA pipes were weighed before the study (T0), on day 7 (T7) and at the end of the study protocol (T14), to evaluate the adherence to treatment. Volunteers who consumed more than 5 g of the product during treatment were considered as adherents.

The 7-point hedonic scale, adapted from Dutcoscky [28], was used to analyze the acceptability of the products. Adverse events reported by volunteers were noted to assess the safety.

### 2.4. Outcomes

The primary outcome was fairness in the clinical cure rate, and, for the secondary outcomes, the fungal burden reduction rate was considered to be >50% of the pretreatment value, the combined clinical and microbiological cure rate, and the acceptability of the products in test. The significance level considered was *P* < 0.05.

### 2.5. Sample Size

The sample size was determined according to the formula described by Chow et al. [29] for parallel 2-arm studies with dichotomous outcome. The calculation considered the clinical response rate with inferiority margin of 10%, alpha 0.05 and power of 0.80, and abandonment rate of 15%, resulting In 20 patients per group. Data analysis was done by intention to treat analysis (ITT).

### 2.6. Randomization: Sequence Generation

The volunteers were allocated into the study groups by simple randomization and electronic generation (http://www.graphpad.com/quickcalcs, GraphPad Software, Inc).

### 2.7. Statistical Analysis

All analysis was performed using GraphPad Prism. The Student *t*-test and Welch correction were used to analyze the quantitative data (age, product intake, time of RD use, and fungal burden). Fisher's exact test was performed for the sexes, and the Mann–Whitney test was used for hygiene frequency. The clinical evolution of the lesions was measured by the Newton's score and compared by the Friedman test with Dunn post-test. The CFU/mL reduction after logarithmic transformation was analyzed by ANOVA for repeated measurements. Clinical and microbiological outcomes were analyzed by Fisher's exact test. The acceptability of the product was compared by the Mann–Whitney test. Descriptive statistics were used for the distribution of* Candida *species.

## 3. Results

**Figure 1:**
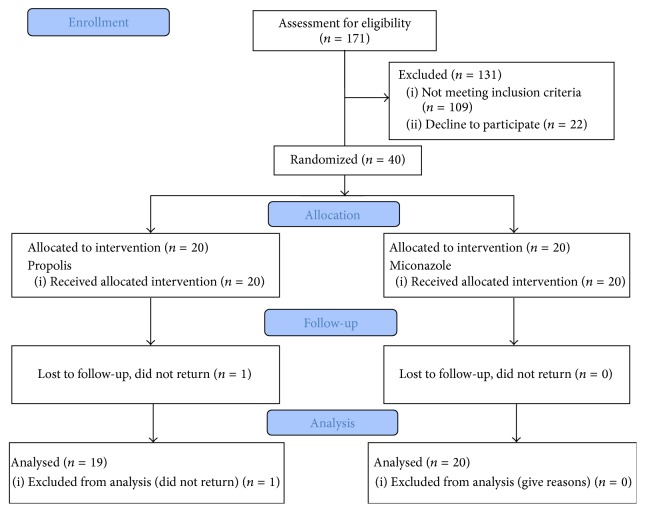
CONSORT flowchart.

Twenty volunteers were randomly allocated to each group, and one volunteer, of the PROP group, lost the follow-up. Volunteers were recruited and examined from January to June 2016. The CONSORT flowchart is shown in Figure 1.  Table 1 summarizes group characteristics, lesion classification, and fungal load. The groups presented female predominance, and no significant differences between the other characteristics were observed. Ninety-five percent of the volunteers reported sleeping with the dentures during nocturnal sleep.

**Figure 2:**
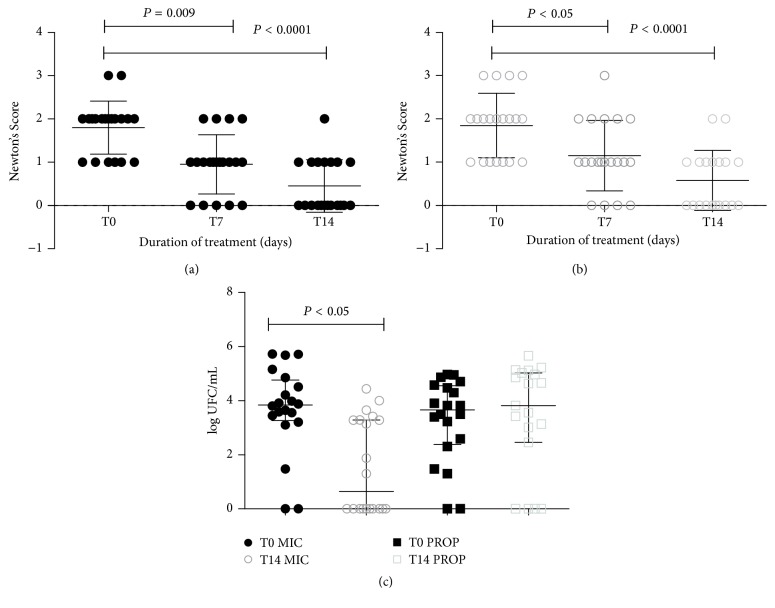
(a) and (b) Clinical evolution measured by Newton's classification ((a) miconazole and (b) EPP-AF). (c) Microbiological evolution measured by colony forming units CFU/mL (c). (a) and (b) Friedman test with Dunn post-test. (c) ANOVA test. The bars represent the median. CI = 95% .

Figure 2 shows the clinical evolution represented by the Newton's classification and the microbiological evolution, as measured by the count of colony forming units. The volunteers majority presented remission of the lesions during the 14 days of treatment and 70% clinical cure rate for both groups. The MIC group, but not the PROP group, presented a significant reduction of the CFU/mL count (*P* = 0.01) in T14.

**Figure 3:**
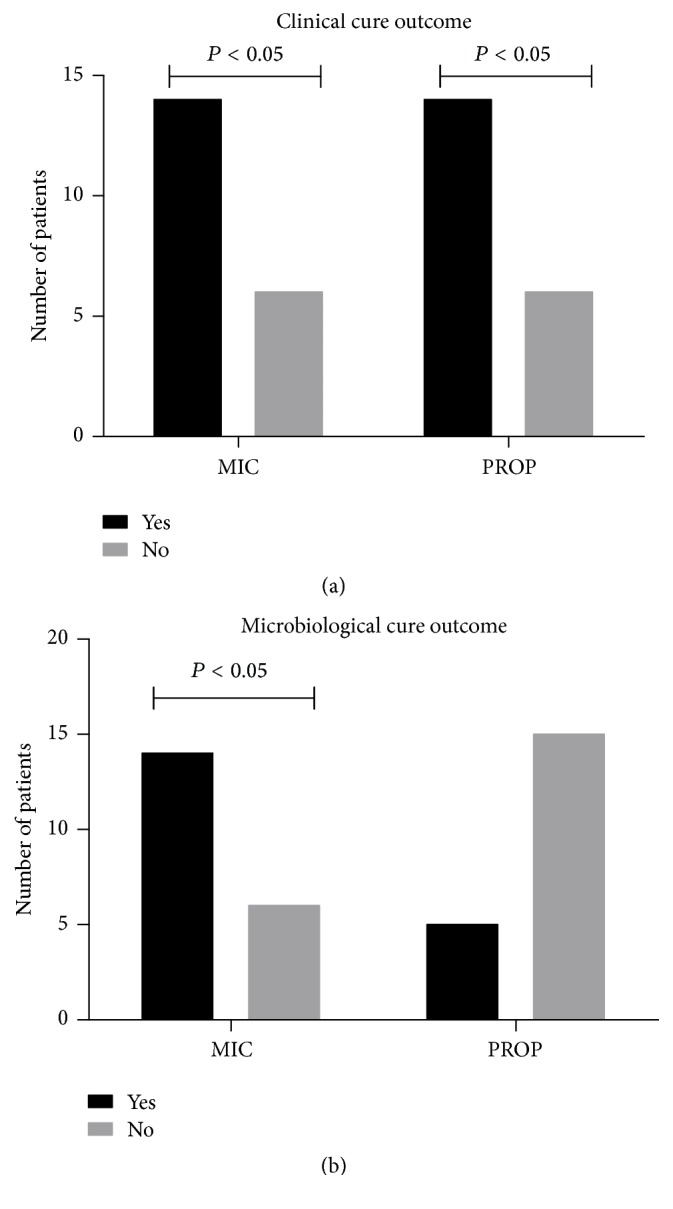
(a) Clinical and (b) microbiological outcomes. The bar chart compares the clinical outcome (a) of the two groups by the clinical improvement observed by Newton's classification and microbiological outcome (b) by the reduction of CFU/mL. (a,b) Fischer's exact test.

The clinical and microbiological outcomes are shown in Figure 3. Both groups showed clinical cure of the lesions in 14 patients (T14). About 14 patients in the MIC group presented reduction of the fungal burden, with microbiological cure, against 5 patients of the PROP group at T14.

In both groups, there was high acceptability to the products used. Ninety-five percent of patients in the PROP group reported having great or very high taste, compared to 70% in the MIC group. Only 5% of the MIC group classified the drug among the scores 1–3 of the hedonic scale, and no patient in the PROP group rated it within this score range. In PROP group, one patient reported burning sensation during application, and no adverse effects were reported by the volunteers in the MIC group.

The* Candida* species identification was performed by the CHROMagar* Candida*. The most prevalent species were* C. albicans* in 90% of the MIC group and 85% of the propolis group. Suggestive species of* C. glabrata* or* C. parapsilosis* were present in 45% of patients in the MIC group and 40% in the PROP group.* Candida tropicalis* appeared in 45% of the PROP group and 20% of the MIC group. Only one patient in each group presented* C. krusei.* Isolated cultures of a single species were observed in 55% and 40% of patients in the MIC and PROP groups, respectively. Cultures with two and three species appear in the MIC group in 30% and 15% and in the PROP group in 40% and 20% in the T0, and in the MIC T14 group, only 5% of the patients had multiple cultures. In the last examination for the PROP group, a reduction of 5% of patients with two or three* Candida* species was observed.

## 4. Discussion

Among the 171 patients examined, 131 were excluded because they did not present lesions of denture stomatitis or did not meet the inclusion criteria of the minimum age. Of the 40 patients analyzed, 85% were female. This data corroborates the literature showing a higher prevalence of denture stomatitis among women [8, 21, 27], possibly due to women's increased demand for treatment or greater health care than men, in addition to having a higher life expectancy compared to this group [30, 31]. All 5 male volunteers have been using a prosthesis over 20 years old and slept with the prosthesis. Also, a hormonal factor may be associated with a female predominance. Golecka-Bakowska and colleagues found that middle-aged women using hormonal supplements and removable total dentures are more prone to the development of oral candidiasis associated with prosthesis use [32].

The analyzed variables are directly related to the increased risk of denture stomatitis. The mean time of use of the current PTRs was 11 ± 9 years for the MIC group and 15 ± 15 years for the PROP group. Although there is no consensus in the current literature regarding the time limit for the use of the same RD [33], there is a significant relationship between the time of use of prosthesis and oral candidiasis. In a study to evaluate the time of use and quality of complete dentures [34], the authors concluded that the time of use influences the overall quality of the prostheses, but it is hard to generalize the useful life in a period of 1 to 10 years.

It is known that, in addition to adhering to oral structures,* Candida* sp. species also colonize the irregularities present in the acrylic structure of the dentures [2, 8, 27, 35]. In our study, thirty-eight patients reported sleeping with the dentures, among a total of forty. This habit is harmful because the contact of the contaminated surface of the denture with the palatal mucosa is continuous, favoring the reduction of salivary flow and the development of denture stomatitis [2, 8, 36–38]. Studies show that only the drug treatment of the patient is not effective, and disinfection of the denture surface is necessary. The association of mechanical method, brushing, and chemical method, astringent solutions and detergents is described as more effective in cleaning and reducing microorganisms [8, 37, 39, 40]. The 0.12% chlorhexidine digluconate spray was used to decontaminate the prosthesis to avoid reinfection by the adhered microorganisms. The antimicrobial potential of chlorhexidine has already been published in the literature showing its activity in biofilm removal and prevention of colonization of* Candida albicans* [21, 36, 41, 42], but its activity depends on an exposure time greater than 30 minutes [42]. Some studies point to an improvement in the appearance of the patient's mucosa with prosthetic stomatitis after local use of chlorhexidine [43, 44]. However, even a short time of exposure to the antiseptic may cause interference in the interpretation of clinical improvement of the lesions.

In our study, older people, in general, were in good health. Some patients presented type II diabetes, considered an immunosuppressive condition when not under control [45] and it was considered a possible confounding factor. However, in addition to this condition being more associated with other types of candidiasis, such as acute and chronic atrophic hyperplasia [1, 2], the number of patients was small, and denture stomatitis is characterized by superficial lesions [46]; therefore, we believe that the diabetes presence did not interfere with the results.

Regarding the prevalence of species, our findings corroborate studies that showed* Candida *albicans as the primary etiological agent of denture stomatitis [2, 21, 27, 36, 47–49].

Previous studies in vitro and in vivo models have demonstrated the absence of cytotoxic and mutagenic potential of propolis gel [12, 22]. Based on these previous studies, the composition used in the present study was determined. The product was formulated with 2.85x the fungicidal concentration obtained in the anti-*Candida albicans* model in vitro, which was 0.7% (7.0 mg/g). Histopathological studies were performed demonstrating that up to 3.6% of propolis did not cause an inflammatory or irritant process [17].

In the present study, the anti-inflammatory and healing action of the formulation based on the propolis extract overlapped the antifungal action, considering that there was a clinical cure, but without reducing the fungal load. The anti-inflammatory action was attributed to the clinical improvement of the lesions. These findings corroborate the research by Berretta et al. [17], which evaluated the healing action of propolis gel on wounds in the epithelium of rats and concluded that concentrations of 2.4% and 3.6% were effective for tissue reconstitution. Some authors demonstrated antifungal activity of propolis, even in small concentrations, and concluded that the antifungal effect was directly proportional to the concentration of propolis. In our study, the concentration of propolis was 2% and incapable of provoking irritant changes to the mucosa according to literature [50]. Therefore, it may be questioned whether the treatment duration or use of higher concentrations could interfere with the antifungal action. Capistrano et al. [19] obtained clinical improvement in prosthetic stomatitis lesions, but without total remission of the lesions in all patients, in a study with a methodology similar to the present study.

We can list important advantages in a natural product with anti-inflammatory, healing and clinical improvement capacity in denture stomatitis when compared to an antifungal drug.* Candida* is a commensal microorganism present in most of the population, so we should consider that its elimination possibly causes an imbalance of the oral microbiome. No pattern was observed between CFU reduction and clinical improvement of denture stomatitis. Volunteers with a high number of colonies presented clinical regression of the lesion, and some patients, who showed little inflammatory regression, presented a reduction in the number of CFUs. One hypothesis could involve the propolis' ability to act on the dimorphism of* Candida albicans* between the pseudohyphae and hyphae forms in yeasts, the latter being nonpathogenic. It is known that this change may be related to the virulence and pathogenicity of the fungus [3, 51–53] with the pseudohyphae and hyphae being the pathogenic morphotypes. Propolis may cause a change in the phenotype of the fungus without necessarily reducing it quantitatively. In prospects for further studies, it should be evaluated the relationship between the clinical improvement of denture stomatitis lesion by EPP-AF treatment and the change in fungus phenotype. Another thing to keep in mind is that propolis also has anti-inflammatory action via the immune system [14] and that candidiasis is triggered, among other possible factors, by an imbalance of this response (immunosuppression) that favors the dimorphic transition to the pathogenic form [1]. Thus, if propolis is acting in the balance of the immune response, it is possible that as a consequence* Candida* transits to the nonpathogenic morphotype; that is, it is not possible to know from the data available at the time if the propolis is acting directly on the causative agent or the host's immune response. Future studies will try to answer these questions. In any case, the remission of clinical symptomatology without the imbalance of the microbiological environment is a fascinating and positive fact.

The acceptability of the product is important for patient adherence to treatment. The nonadherent patient may have difficulties in maintaining dosage and may suffer from inadequate exposure to antifungal, adverse effects [54], a risk of drug interactions [55–57], and microorganism resistance, commonly observed in azole antifungals [10, 11, 58]. Miconazole acts by altering the cell membrane permeability and is considered the antifungal of choice for topical treatment of denture stomatitis in healthy patients. However, its chronic use may cause the resistance of* Candida* species to miconazole in elderly patients with denture stomatitis [10, 59]. Results of a meta-analysis showed that miconazole showed greater clinical efficacy for the treatment of denture stomatitis compared to natural substances, including propolis [54]. However, in our study, clinical improvement was similar between the two products. Miconazole, in spite of its local action, may be sufficiently absorbed to the point of an interaction with other drugs. There are reports of interaction between warfarin, in addition to antidepressants, antihistamines, immunosuppressants, antiepileptics, and miconazole oral gel [55, 56]. The authors still caution against the consideration of interactions between drugs by dental surgeons before prescriptions.

Some studies indicate that patients exposed to risk factors and presenting recurrent candidiasis should be treated using antifungals with lower potential for developing resistance in microorganisms [6, 38]. Regarding this issue, propolis demonstrated antifungal action by at least three mechanisms of action in the model of deletion strains of* S. cerevisiae *[18, 60] and* C. albicans* [61], making it difficult to develop microbiological resistance.

The data here have shown an anti-inflammatory and cicatrizing action on the dentures stomatitis by the EPP-AF mucoadhesive oral gel formulation. Thus, the use of this formulation could decrease the time of treatment and antifungal exposure, which could reduce the risk of fungal resistance using EPP-AF, isolated or in association with commercial antimicrobials. The elderly are a target audience for the development of repetitive denture stomatitis, due to the high rate of use of removable dentures. Therefore, the ideal treatment is effective alternatives and with minimal risk, offering greater safety. For future studies, the microbiological evaluation of phenotypic and genotypic characteristics as possible correlations between* Candida *species and the degree of clinical improvement of the lesions is suggested, as well as the evaluation of immune response on the region of stomatitis lesions in response to both treatments.

## 5. Conclusions

The EPP-AF appears to be noninferior to miconazole considering the clinical cure rate of denture stomatitis in older adults and could be recommended as an alternative and complementary treatment for these patients.

## Figures and Tables

**Table 1:** Baseline characteristics of the study groups, classification of palatine lesions, and initial fungal burden.

Variable	MIC (*n* = 20)	PROP (*n* = 20)	*P* value
Age (years)	73 ± 9	73 ± 7	0,98
Gender (F%)	80	90	0,66
RD: time of use (years)	30 ± 13	35 ± 11	0,20
Current: RD time of use (years)	11 ± 9	15 ± 15	0,23
Frequency of RD hygiene (times/day)	3 (2-3)	3 (3-3)	0,31
Newton's score (T0)	2 (1-2)	2 (1-2)	0,98
Amount of gel (g)	17 ± 13	20 ± 17	0,55
Unity count (log CFU/mL) T0	3,7 ± 1,6	3,3 ± 1,6	0,44
Diabetics	4	2	0,66

Data expressed as mean (median) and SD (range 25 to 75% range).
